# West Nile virus surveillance in Europe: moving towards an integrated animal-human-vector approach

**DOI:** 10.2807/1560-7917.ES.2017.22.18.30526

**Published:** 2017-05-04

**Authors:** Céline M Gossner, Laurence Marrama, Marianne Carson, Franz Allerberger, Paolo Calistri, Dimitrios Dilaveris, Sylvie Lecollinet, Dilys Morgan, Norbert Nowotny, Marie-Claire Paty, Danai Pervanidou, Caterina Rizzo, Helen Roberts, Friedrich Schmoll, Wim Van Bortel, Andrea Gervelmeyer

**Affiliations:** 1 Surveillance and Response Support Unit, European Centre for Disease Prevention and Control (ECDC), Stockholm, Sweden; 2 Animal and Plant Health Unit, European Food Safety Authority (EFSA), Parma, Italy; 3 Units for Animal Health and Public Health, Austrian Agency for Health and Food Safety (AGES), Vienna, Austria; 4 Istituto Zooprofilattico Sperimentale dell’Abruzzo e del Molise ‘G. Caporale’, Teramo, Italy; 5 Ministry of Rural Development and Food, Animal Health Directorate, Athens, Greece; 6 French Agency for Food, Environmental and Occupational Health & Safety (ANSES), Animal Health Laboratory, EU-RL on equine diseases, Maisons-Alfort, France; 7 Emerging Infections and Zoonoses, Public Health England, Colindale, United Kingdom; 8 Institute of Virology, University of Veterinary Medicine, Vienna, Austria; 9 Department of Basic Medical Sciences, College of Medicine, Mohammed Bin Rashid University of Medicine and Health Sciences, Dubai, United Arab Emirates; 10 Santé publique France, Saint Maurice, France; 11 Hellenic Center for Disease Control & Prevention, Department of Epidemiological Surveillance and Intervention, Vector-borne Diseases Office, Athens, Greece; 12 Istituto Superiore di Sanità (ISS), Rome, Italy; 13 Veterinary and Science Policy Advice team, Animal and Plant Health Agency, Weybridge, United Kingdom

**Keywords:** West Nile fever, West Nile virus, surveillance, one health, European Union, Collaboration

## Abstract

This article uses the experience of five European countries to review the integrated approaches (human, animal and vector) for surveillance and monitoring of West Nile virus (WNV) at national and European levels. The epidemiological situation of West Nile fever in Europe is heterogeneous. No model of surveillance and monitoring fits all, hence this article merely encourages countries to implement the integrated approach that meets their needs. Integration of surveillance and monitoring activities conducted by the public health authorities, the animal health authorities and the authorities in charge of vector surveillance and control should improve efficiency and save resources by implementing targeted measures. The creation of a formal interagency working group is identified as a crucial step towards integration. Blood safety is a key incentive for public health authorities to allocate sufficient resources for WNV surveillance, while the facts that an effective vaccine is available for horses and that most infected animals remain asymptomatic make the disease a lesser priority for animal health authorities. The examples described here can support other European countries wishing to strengthen their WNV surveillance or preparedness, and also serve as a model for surveillance and monitoring of other (vector-borne) zoonotic infections.

## Introduction

West Nile fever (WNF) is a zoonotic vector-borne disease caused by a virus that is most often transmitted through mosquito bites (primarily *Culex* genus) but can also be transmitted through organ transplantation, blood transfusion, in laboratory settings and from mother to fetus during pregnancy [1]. West Nile virus (WNV) is maintained in a bird-mosquito cycle, with birds acting as amplifying hosts. Mosquitoes acquire infection by feeding on viraemic birds. Once infected, the mosquito remains infectious throughout its life, potentially transmitting the virus to every vertebrate on which it feeds. Many bird species do not develop any disease symptoms after infection. However, certain species, such as crows, jays and birds of prey, may die from the infection [2].

Humans, horses and other mammals are considered dead-end hosts. Infections in humans are generally asymptomatic. Around 20% of cases develop influenza-like symptoms, while 1% of cases, mainly elderly and immunocompromised people, develop West Nile neuroinvasive disease (WNND), which may lead to death [3]. Approximately 10% of infected horses may show neurological signs [4]. There is no specific treatment for humans or animals, and no vaccine is available for humans, although inactivated and recombinant vaccines for horses are used in Europe [5].

The epidemiological situation of WNF in Europe is heterogeneous: some European countries report outbreaks in humans and animals every year and others have never reported any autochthonous cases [6,7]. Taking five European countries (Austria, France, Greece, Italy and the United Kingdom (UK)) with very diverse WNF epidemiological situations as examples, this article describes surveillance and monitoring activities for WNV infection in humans, animals and vectors conducted at national and European levels, and suggests key actions for strengthening the intersectoral collaboration between the public health and veterinary sector.

## Epidemiological situation in Austria, France, Greece, Italy and the United Kingdom

### Austria

In Austria, the first autochthonous human WNF cases were diagnosed retrospectively by serology: two cases from 2009 and one case from 2010 [8] (Table 1).

**Table 1:** Number of humans, horses, birds and mosquito pools tested and found to be infected with West Nile virus in Austria, France, Greece, Italy, and the United Kingdom, 2010–2015

Number of humans^a^, horses, birds and mosquito pools found to be infected with West Nile virus (number of humans, horses, birds and mosquito pools tested)	2010		2011		2012		2013		2014		2015	
	Number infected	Number tested	Number infected	Number tested	Number infected	Number tested	Number infected	Number tested	Number infected	Number tested	Number infected	Number tested
**Austria**												
Human cases	1	NA	0	NA	0	NA	0	NA	2	NA	8	NA
Equine cases	0	NA	0	NA	0	NA	0	NA	0	NA	0	NA
Positive birds	0	NA	0	NA	0	NA	1	NA	1	NA	2	NA
Positive mosquito pools	0	NA	1	NA	1	NA	0	NA	2	NA	3	NA
**France **												
Human cases	0	NA	0	NA	0	NA	0	NA	0	NA	1	NA
Equine cases	0	94	0	85	0	67	0	54	0	39	49	155
Positive birds	0	4	0	0	0	5	0	9	0	0	0	15
Positive mosquito pools	NA	NA	NA	NA	NA	NA	NA	NA	NA	NA	1	40
**Greece **												
Human cases	262	NA	100	NA	161	NA	86	NA	15	NA	0	NA
Equine cases	30	167	24	1,539	15	1,640	15	1,626	4	962	0	NA
Positive birds	NA	NA	NA	NA	NA	NA	NA	NA	NA	NA	NA	NA
Positive mosquito pools	2	110	71	897	212	2,112	45	405	6	603	11	157
**Italy **												
Human cases	3	NA	14	NA	28	NA	44	NA	21	NA	38	NA
Equine cases	128	993	197	2,840	63	1,343	50	3,366	27	7,675	30	5,507
Positive birds	16	3,614	16	4,719	26	5,363	79	5,649	55	5,018	73	1,880
Positive mosquito pools	13	1,236	8	3,059	14	2,907	146	1,984	125	7,047	102	4,614
**United Kingdom**												
Human cases	0	NA	0	NA	0	NA	0	NA	0	NA	0	NA
Equine cases	0	5	0	10	0	10	0	12	0	5	0	3
Positive birds	0	204	0	280	0	374	0	316	0	433	0	336
Positive mosquito pools	NA	NA	NA	NA	NA	NA	NA	NA	NA	NA	0	NA

NA: not available.

^a^ Includes probable and confirmed autochthonous West Nile neuroinvasive disease (WNND) and non-WNND cases.

In 2014, two more people were affected by WNV [9], followed by eight cases in 2015. WNV was introduced in eastern Austria in 2008 [10] causing mortality in birds. Since then, WNV has been repeatedly found in mosquito pools and birds in Vienna [9], Lower Austria [10] and in regions bordering the Czech Republic [11].

### France

No evidence of WNV infections in humans or horses was identified in France from the mid-1960s until 2000 [12]. The 2000 WNV epizootic among equidae in the Camargue was the largest ever recorded in France although it did not cause massive bird die-offs [12]. In the following years, WNV circulation was reported on three occasions in the Camargue and neighbouring regions [13,14]. Seven autochthonous human cases were reported in 2003 and then none until 2015 (Table 1). Serosurveys conducted in the Camargue in the periods 2005–2007 and 2009–2010 highlighted WNV circulation in resident birds in the absence of cases in humans or horses. However, no formal proof of virus endemicity in the wild avifauna from the Camargue has yet been obtained [15,16]. In summer 2015, WNV re-emerged at the periphery of the Camargue, causing WNND in horses and one WNF human case, reminding us that the Camargue remains a potential environment for WNV circulation.

### Greece

In Greece, the first outbreak of WNF in humans occurred in 2010, in the region of Central Macedonia [17]. Between 2010 and 2014, the virus spread further, with annual seasonal outbreaks recorded in humans and animals, between June and October. During that period, 624 autochthonous human WNF cases were diagnosed in Greece, in 11 of 13 regions, whereas in 2015 no human cases were diagnosed in the country (Table 1). Although neutralising antibodies against WNV had previously been detected in horses [18], the first WNF cases in equidae in Greece were detected in 2010 in Central Macedonia, after the occurrence of the first human cases. Since 2010, the number of affected horses has been decreasing (Table 1).

### Italy

In Italy, WNV reappeared in the north-east of the country in the summer of 2008 after a 20-year absence; WNV was isolated in mosquitoes, birds, equidae and humans in the area surrounding the Po river delta [19,20]. From 2010 to 2015, 148 confirmed autochthonous human cases of WNND were reported in Italy from eight of the 20 regions (Table 1). Seroconversion in horses and sentinel chickens was regularly identified in the wetlands of Sicily during this period, whereas sporadic animal cases have been detected in some localities of central and southern Italy.

### United Kingdom

The UK has not had any autochthonous human or animal cases of WNF, and WNV infection has never been found in vector species there. Although there have been a few limited studies in sentinel chickens and non-migratory wild birds suggesting positive WNV antibody reactions and detection of WNV RNA in avian tissues, these results have never been reproduced and validated [21].

## West Nile virus surveillance and monitoring at European Union level and in the individual countries

The key characteristics of WNV infection surveillance in the European Union (EU) and the individual countries are summarised in Table 2. Notifications of human WNF cases in Europe are collected through The European Surveillance System (TESSy) [22] of the European Centre for Disease Prevention and Control (ECDC). Between June and November, the period of high vector activity, ECDC publishes weekly updated maps [7] of human cases and complementary information on animal WNV infection and vectors based on data provided by the World Organisation for Animal Health (OIE) and European countries. The yearly analyses of TESSy data are published in the ECDC annual epidemiological report [23] and jointly with the European Food Safety Authority (EFSA) [6].

**Table 2:** Key characteristics of West Nile virus (WNV) infection surveillance in the European Union, Austria, France, Greece, Italy and the United Kingdom

Country/region	Intersectoral collaboration	Human surveillance	Animal surveillance	Vector surveillance
**European Union**	▪ ECDC provides WNF seasonal maps that include human cases and provides information on animal/vector WNV infection. ▪ ECDC tool for WNF risk assessment proposes a classification of risk areas based on human, animal and vector surveillance data. ▪ EFSA and ECDC publish the EU summary reports on trends and sources of zoonoses, zoonotic agents and food-borne outbreaks every year.	▪ WNF is an EU notifiable disease; cases are reported by EU countries to TESSy according to the EU case definition. ▪ EU countries must implement a deferral of blood donations for 28 days after leaving an area with ongoing WNV transmission in humans.	▪ WNF, as a cause of equine encephalomyelitis in horses, is notifiable to the European Animal Disease Notification System. ▪ WNF in animals is a disease listed in the OIE Terrestrial Animal Health Code and must be reported to the OIE. ▪ EU countries should monitor WNV activity in animals, if warranted by the epidemiological situation, and report animal cases to the European Commission.	▪ There is no legal framework regarding mosquito surveillance at EU level. ▪ ECDC developed guidelines for surveillance of native mosquitoes to support countries to plan and implement surveillance activities.
**Austria**	▪ A national WNV Task Force was established in 2013, with members nominated by the Ministry of Health. ▪ No joint reporting system across the different authorities. However, reports are published on the AGES website.	▪ Human cases and fatalities are statutorily reportable as of September 2015. ▪ Routine examination of donated blood was introduced in eastern Austria in the summer of 2014.	▪ Neurological disease in equidae is notifiable in Austria. ▪ Surveillance covers birds and horses.	▪ Since 2011, active country-wide mosquito surveillance is conducted by AGES.
**France**	▪ The national guidelines for WNV surveillance, prevention and control activities are under the responsibility of the Ministries of Health, Agriculture and Environment.	▪ Suspected neuroinvasive human cases should be notified to regional health authorities in the Mediterranean region during the period of vector activity, from June to November. ▪ Measures for the safety of blood products are taken in line with the EU directive for blood safety.	▪ Notification of suspected horse cases to regional veterinary services is compulsory, whatever the time period or their location. ▪ Sentinel bird surveillance was discontinued in 2007.	▪ Mosquito surveillance is systematically implemented from March to November in the Mediterranean area.
**Greece**	▪ The Ministry of Rural Development and Food and the HCDCP share the results of human, animal and vector surveillance with each other and with regional and local public health authorities, local veterinary services, municipalities and local health units ▪ There are a multisectoral committee for the prevention and management of tropical diseases (including WNF) and two multisectoral working groups: for vector-borne diseases and for the designation of areas affected by such diseases.	▪ Since 2010, all laboratory probable and confirmed WNF cases should be notified. ▪ Enhanced surveillance is implemented at national level that includes awareness campaigns towards physicians, support of laboratory confirmation, active laboratory-based surveillance, cases investigation and daily dissemination of information to national and local stakeholders. ▪ Measures for the safety of blood products are taken in line with the EU directive for blood safety.	▪ WNF in animals is a notifiable disease and disease suspicions must be reported to the competent veterinary authorities. ▪ There is active serological surveillance of sentinel horses; active clinical surveillance of equidae around confirmed human and animal cases; passive surveillance of WNF in equidae all-year-round and some small scale surveillance in wild birds.	▪ Since 2010, HCDCP together with the National School of Public Health, Universities, local authorities and subcontractors, conducts active vector surveillance from June to October to detect WNV circulation in mosquitoes.
**Italy**	▪ A national plan for surveillance on imported and autochthonous human vector-borne disease (chikungunya, dengue and WND) integrating human and veterinary surveillance is prepared annually. ▪ Any suspected evidence of virus circulation in animals or vectors is notified to the public health authorities.	▪ Probable and confirmed human cases are notified in real time using a password-protected web-based system. ▪ In the affected area, local health authorities implement an active surveillance at risk population. ▪ Passive surveillance on human neurological cases is set up in the whole region where the affected area is located. ▪ Measures for the safety of blood products are taken in line with the EU directive for blood safety.	▪ A web-based national animal disease notification system allows the notification of animal diseases. ▪ Passive surveillance in equidae in the whole country. ▪ Random IgM screening in horses living in non-affected areas. ▪ Bird surveillance focuses on WNV detection in resident target species; immunological response among poultry of rural farms and migratory birds; and bird mortality.	▪ Entomological surveillance is systematically implemented during the period of vector activity in affected areas.
**United Kingdom**	▪ A national contingency plan for integrated surveillance and control of vector-borne disease has been prepared. ▪ Monthly meetings between human and animal health risk assessors to share information. ▪ Any suspect or confirmed cases would be notified to all relevant authorities.	▪ WNV in humans is a notifiable disease in Scotland, but not in the rest of the UK. It is however, a notifiable organism in England, Wales and Scotland (not Northern Ireland). ▪ Human cases of autochthonous WNV infection should be reported to National Surveillance Centres by the diagnostic laboratories. ▪ Measures for the safety of blood products are taken in line with the EU directive for blood safety.	▪ WNF, as a cause of equine encephalomyelitis, is a notifiable disease of equidae in the UK. ▪ There is no active surveillance of horses or other equidae. Monitoring relies on passive surveillance and testing of horses with neurological signs. ▪ There is no systematic WNV active surveillance of wild birds in the UK, however a passive surveillance system is in place between April and October.	▪ Some targeted surveillance for mosquitoes are carried out by PHE in areas with suitable habitat.

AGES: Austrian Agency for Health and Food Safety; ECDC: European Centre for Disease Prevention and Control; EFSA: The European Food Safety Agency; EU: European Union; HCDCP: Hellenic Center for Disease Control and Prevention; OIE: World Organization for Animal Health; PHE: Public Health England; TESSY: The European Surveillance System; UK: United Kingdom; WNF: West Nile fever; WNV: West Nile virus.

EU countries report outbreaks of WNV encephalomyelitis in horses to the European Commission (EC) via the Animal Disease Notification System (ADNS) [24] and regular summaries are posted online. The data from WNV monitoring in animals is reported annually by EU countries under Directive 2003/99/EC [25] and presented in the annual EFSA/ECDC EU Summary Report on Trends and Sources of Zoonoses, Zoonotic Agents and Food-borne Outbreaks [6]. Animal WNF outbreak data reported to the OIE are publically available on the World Animal Health Information Database (WAHIS) interface [26].

Through the Pan-European VectorNet project [27], presence/absence distribution maps of *Culex* species are under development. Current *Culex pipiens* maps are incomplete [28].

### Austria

The Austrian Federal Ministry of Health has developed WNV guidelines for the Austrian Blood Donation System, based on the document ‘West Nile Virus and Blood Safety - Introduction to a Preparedness Plan in Europe’ [29]. In response to the blood donation findings in 2014, three eastern Austrian federal states switched to pooled testing of blood donations.

Veterinary surveillance of WNV in Austria covers birds and horses. Bird surveillance has been carried out since 2008. Screening is conducted in all cases of encephalitis in birds with emphasis on *Falconiformes* and *Passeriformes*; active surveillance of birds sampled under the avian influenza monitoring programme/scheme is conducted by serological testing of waterfowl (geese, ducks) sampled every year from slaughterhouses in at-risk regions. All suspected encephalomyelitis cases in horses must be notified and are tested for WNV; the first WNV-induced case of equine encephalitis was documented in September 2016. Also, a national serological screening programme for WNV antibodies in horses has been in place since 2011.

Since 2011, the Austrian Agency for Health and Food Safety (AGES) [30] has conducted active country-wide mosquito surveillance. This involves mosquito species identification and laboratory testing for various pathogens including WNV at two sampling sites per province. Any human or animal WND case leads to enhanced vector surveillance in the respective area.

A national WNV Task Force was established in 2013, with members nominated by the Ministry of Health. This Task Force brings together representatives from all affected provinces as well as those responsible for vector surveillance/control, public and animal health. The group meets at least once a year, and more frequently when WNF cases are identified. A similar federal group already exists for investigations of outbreaks of food-borne and other zoonoses.

Austria does not have a joint reporting system across the different authorities. However, reports are published on the AGES website, including maps containing compiled information on human and animal cases as well as the results of the mosquito surveillance. Detailed reports (other than the general reports published on the AGES website) are produced exclusively for the use of the health authorities. The public is informed about WNF cases through press releases.

### France

At national level, human surveillance activities include the notification of confirmed human cases by the National Reference Centre, Marseille. Enhanced surveillance of human neuroinvasive cases is implemented in the Mediterranean region between June and October. Surveillance of human non-neuroinvasive cases is conducted by the National Reference Centre and Santé Publique France.

Clinical surveillance in equidae is carried out across the entire country, with veterinary practitioners reporting suspected cases to regional veterinary services. Established in 1999, the French network Réseau d’Epidémio-Surveillance en Pathologie Equine (RESPE) supports 550 sentinel and voluntary veterinarians across France in testing symptomatic horses for the identification of causative agents for diverse conditions (e.g. neurological infections, acute respiratory infections), which includes WNV. Periodic reports on the WNV situation in horses during WNV outbreaks are made available on the online platform Epidémiosurveillance santé animale (ESA) [31].

Sentinel bird surveillance was discontinued in 2007, with the option to reactivate it should the epidemiological context change. Clinical bird surveillance, relying on WNV testing of abnormal bird fatalities from June to October in the Mediterranean area, was combined with avian influenza surveillance in 2006. The sensitivity of this bird surveillance is however very low as screening is performed on a very small subset of dead birds.

WNV screening in mosquitoes is no longer conducted outside WNV epizootics or epidemics. However, mosquito surveillance, involving the identification of mosquito species and abundance, has been systematically implemented from March to November in the Mediterranean area by the Interdepartmental Agreement for Mosquito Control on the Mediterranean coast.

WNV surveillance, prevention and control activities are described in national guidelines that were published for the first time in 2004, last updated in 2012, under the responsibility of the Ministries of Health, Agriculture and Environment. Blood safety measures described in these guidelines are in line with the EU directive [32].

### Greece

Human surveillance includes awareness campaigns among physicians, support of laboratory confirmation and active laboratory-based surveillance with daily exchange of information on the diagnosed cases between the Hellenic Center for Disease Control and Prevention (HCDCP) and the laboratories testing for WNV. All probable and confirmed cases are investigated within 24 hours after diagnosis by HCDCP and there is a daily follow-up of all hospitalised cases until discharge. National and local stakeholders, including the blood safety authorities, receive daily updates on diagnosed cases and weekly surveillance reports by email, the latter also being publically available on the HCDCP website. Affected areas are defined as third administrative level areas with at least one human case.

In line with the EC directives 2004/33 [32] and 2014/110 [33] and the EC WNV and blood safety preparedness plan [29], blood safety measures have been implemented in affected areas, including screening of donor blood for WNV RNA.

Since 2010, the HCDCP’s Coordinating Haemovigilance Centre has conducted active surveillance of WNV infection in the blood donor population of the affected areas during the transmission period from mid-June to mid-November. All confirmed cases of WNV infection in blood donors are notified to the Haemovigilance Centre. Specific haemovigilance procedures such as post-donation and post-transfusion information are in place: When a case of WNV infection reports a recent blood transfusion, trace back and testing of the implicated donors is initiated. Moreover, blood donors are asked to notify any suspected symptom within 15 days after donation and if they do, blood testing is performed [34].

Systematic surveillance for the most important diseases of equidae, including WNF, had previously been in place in Greece (2001–2004), carried out by the local veterinary authorities, under the coordination of the central veterinary service [18]. Since 2010, a WNF-specific surveillance programme has been implemented under the coordination of the Ministry of Rural Development and Food in cooperation with the local veterinary authorities and relevant state laboratories within the Veterinary Centres of Athens and Thessaloniki (Ministry of Rural Development and Food). The programme includes active serological surveillance of sentinel horses; active clinical surveillance of equidae around confirmed human and animal cases; passive surveillance of WNF in equidae all year round; passive surveillance of wild birds by sampling dead or sick wild birds; and active surveillance of wild birds through capture and sampling in selected regions. In addition, schools of veterinary medicine perform WNV surveys in domestic and wild birds in various areas.

Since 2010, HCDCP together with the National School of Public Health, Universities, local authorities and subcontractors have conducted active vector surveillance from June to October, including WNV detection in mosquitoes [35].

The Ministry of Rural Development and Food and the HCDCP share the results of animal and vector surveillance with regional and local public health authorities, local veterinary services, municipalities and local health units for further awareness, prevention and follow-up activities.

There is a multisectoral committee for the prevention and management of tropical diseases (including WNF) of the Ministry of Health, and two multisectoral working groups: for vector-borne diseases and for the designation of areas affected by such diseases. These groups ensure communication between veterinary and human health authorities, entomologists, blood safety authorities, infectious disease specialists and other national actors.

### Italy

In Italy, public and animal health surveillance is a shared responsibility between the national and regional levels.

The national plan for human surveillance defines ‘affected areas’ as all the provinces (NUTS-3 level) where laboratory-confirmed WNV infections in animals, vectors or humans have been notified in the previous year or during the current surveillance period (between 15 June and 30 November, the period considered to have the highest vector activity). In the affected area, local health authorities implement active surveillance in employees of farms where equine cases are identified and in individuals living or working in the surrounding area. Employees of affected farms are contacted regularly by phone and serosurveys are conducted. Moreover, measures for vector control and blood and transplant safety are implemented immediately. At the same time, passive surveillance of human WNND cases is undertaken in the whole region in which the affected area is located, requesting physicians to report all probable and confirmed WNND cases using a modified EU case definition, which includes neurological symptoms.

Probable and confirmed human cases are notified by regional authorities to the Ministry of Health and to the Istituto Superiore di Sanità using a password-protected web-based system gathering epidemiological and laboratory information about cases. The database is also accessible by the National Blood Centre and the National Transplant Network in order to implement measures on blood and transplants safety in a timely manner. Since 2013 all asymptomatic confirmed cases of WNV infection in blood donors have also been notified through the web-based system.

The surveillance activities for wild birds and vectors have been strengthened since 2013. Bird surveillance focuses on three aspects: WNV detection in resident target species such as magpies and crows; immunological response among poultry of rural farms, sentinel chickens and migratory birds; and bird mortality. Entomological surveillance includes WNV detection in mosquito pools from affected areas.

There is passive surveillance in equidae with random serological tests in non-endemic areas and monitoring of sentinel horses. However, many horses in Italy are vaccinated against WNV.

A web-based national animal disease notification system allows the notification of animal diseases. The system allows the integration of data from veterinary field services and laboratories into a national database. The database is accessible by different national stakeholders, including the Ministry of Health and the National Blood Centre, and weekly reports are published online [36,37].

### United Kingdom

Surveillance activities targeting humans, animals and vector sources have been in place since 2002. Human cases of autochthonous WNV infection should be reported to National Surveillance Centres by the diagnostic laboratories as a matter of urgency. However, the causative organism of just over a third of cases of encephalitis remains undiagnosed [38].

Safeguards are in place to protect the UK blood supply from WNV. These include deferring donation for 28 days from the date of leaving a WNV-affected area, unless WNV nucleic acid test screening is in place to maintain a sufficient blood supply [39].

There is no active surveillance of horses or other equidae. Monitoring relies on passive surveillance and testing of horses with neurological signs. The Animal Health Trust has the responsibility for equine health under a contract from the Department for Environment Food and Rural Affairs (Defra). Syndromic surveillance is carried out and all suspected WNF cases must be reported to the Animal and Plant Health Agency (APHA). In addition, there is an option for private veterinary surgeons (PVS) to submit samples for testing for WNV as a differential diagnosis, which will not trigger an official investigation, as the probability of diagnosis would be considered low. The PVS must discuss the clinical and import history of the horse with APHA, and if WNV is low on the list of differential diagnoses and the owner still requests a test to rule out infection, samples may be taken at the owner’s expense. If there is suspicion of a notifiable disease, this will trigger a disease investigation. All samples must be tested by a UK reference laboratory for WNV.

APHA is responsible for testing WNV-target wild birds (e.g. small *Passeriformes*, corvids and waterside birds) found dead. There is no systematic WNV active surveillance of wild birds in the UK, but a passive surveillance system is in place between April and October. For any of the target wild bird species, birds with neurological signs or large die-offs, that are reported to the Defra helpline or via a warden patrol at specified wild bird reserves, will be collected and tested for a range of avian diseases, which include WNV. Approximately 300 to 400 birds are tested each year.

There is no formal programme for year-round country-wide vector surveillance in the UK. Instead, some targeted surveillance for mosquitoes is carried out by Public Health England (national public health agency) in areas with suitable habitat, to monitor the distribution and abundance of WNV vector species and test for WNV infection. As a result, *Culex modestus* was identified in two counties in the Thames estuary, suggesting that the vector has been endemic in this area since 2010 [40].

## Discussion

### Surveillance strategies adapted to the countries’ epidemiological situations

Human surveillance in Austria, France, Greece and Italy targets early detection of WNV infection cases, and identification of affected areas to implement appropriate response measures: including blood safety measures, vector control and communication to relevant authorities and to the public (Table 2).

In the long term, it aims to quantify the disease burden, identifying seasonal, geographic and demographic patterns of morbidity and mortality. In the UK, where no autochthonous cases of WNV infection have ever been detected, human surveillance focuses on preparedness.

While serosurveys in horses can be used to determine absence/presence of WNV circulation in an area where no or limited data are available, the utility of serosurveys’ results is limited by the background level of immunity (acquired by natural infection or by vaccination) of the population. Infection in horses may occur at the same time or even later than the identification of the first human cases [41]. The usefulness of WNV surveillance in equidae for early detection purposes is generally considered to be limited but in countries with irregular WNV outbreaks such as France, the screening of horses is considered by the French authorities to be of added value. Considering that more and more horse owners in affected countries are vaccinating their horses, it is estimated that surveillance in equidae will gradually become irrelevant.

Surveillance of birds and mosquitoes aims at early detection of WNV circulation at the beginning of a new vector season and the identification of areas of virus circulation. This surveillance is used to promptly inform public health authorities. Sentinel domestic birds are easily exposed to the mosquito fauna, handled and monitored over several months, making them suitable targets for WNV surveillance. In domestic pigeons (*Columba livia domestica*) and free-ranging chickens under 5 months old WNV circulation has been detected more than a month before the onset of an epidemic in humans [42,43]. The detection of WNV RNA in mosquitoes has resulted in detection of WNV circulation ca 2–5 weeks earlier than serological monitoring of sentinel chickens at equal spatial sampling density [44]. The downside of surveillance in captive sentinel birds and in mosquitoes are the high costs and logistical demands, which make these surveillance options cost effective only for countries that have regular large outbreaks such as Italy.

Countries free of WNV, like the UK, can achieve early warning of increased risk of WNV introduction through suitability mapping; i.e. spatial analytic studies of WNV risk predictors based on the combination of animal, human and environmental data from their own country and neighbouring countries, to identify areas at risk for WNF outbreak should the virus be introduced [45].

There is no one-fits-all surveillance strategy. Each country needs to assess its epidemiological situation and local conditions to identify the integrated approach that best meets its needs.

### An integrated approach for West Nile virus surveillance

Surveillance activities are considered integrated when they are coordinated jointly by public health authorities, animal health authorities and authorities in charge of vector surveillance and control, with the aim of reaching a common understanding of threat level and disease activity. Such integration is expected to improve surveillance and monitoring efficiency and to save resources by implementing targeted measures. An integrated approach requires regular exchanges of information between all actors. In order to improve collaboration, the creation of a formal interagency working group supported by the respective ministries was found to be a crucial step (i.e. in Austria, France, Greece, Italy and the UK). Regular meetings, timely sharing of data among the working-group members and the development of a joint information exchange platform are instrumental.

An integrated collection and analysis of data from human, animal and vector surveillance, ideally in a single database, is key to obtain a comprehensive understanding of the epidemiological situation of WNV and consequently timely implement response measures. The modalities of the integrated approach are country-dependent taking into account the local context.

### Challenges of the integrated approach and avenues to strengthen intersectoral collaboration

WNF is a notifiable disease in humans and in equidae at the EU level. While for public health ensuring blood safety is a key incentive to allocate sufficient resources for WNF surveillance, on the animal side, the facts that an effective vaccine is available and that most cases remain asymptomatic make the disease less of a priority. In Europe, expertise and resources in vector surveillance and control are variable but not always well integrated with the public and veterinary health sectors. Development of in-country entomology expertise and the provision of sufficient funding are key to develop adequate vector surveillance and control capacities.

The role of wild animals in the WNV transmission cycle makes the implementation of control measures challenging. In addition, as multiple stakeholders from the public health sector, the animal health sector and the environmental sector are involved in the implementation of control measures, coordination becomes complex if there are no clear guidelines and established collaboration arrangements. Setting clear common objectives can overcome some of these challenges and will allow joining resources and expertise.

## Conclusions

WNV surveillance is challenging as the virus transmission cycle is complex and most human and animal cases remain asymptomatic, which poses a risk for transmission by blood products. An integrated approach, involving public health, animal health and environmental authorities offers the most efficient and effective mechanism for tackling WNV transmission. Austria, France, Greece, Italy and the UK have implemented different surveillance strategies tailored to their epidemiological situation with different degrees of integration across disciplines and authorities. The examples described here can support other European countries wishing to strengthen their WNV surveillance or preparedness plans and serve as examples for surveillance and monitoring of other (vector-borne) zoonotic infections. The example from the UK shows that even without the presence of the disease an integrated plan can support preparedness.
